# Supplementary material for: Urine-derived exosomes from individuals with IPF carry pro-fibrotic cargo
Source: eLife. 2022 Dec 1;11:e79543. doi: 10.7554/eLife.79543 (PMC9714968; doi:10.7554/eLife.79543)
Supplement: Source data 1. [file elife-79543-data1.pdf]

<u>Supplement 1 source data</u>: Primers (either sequence or catalog numbers), antibodies

**Primer details (ABI)**		**Gene Expression number**	
**RNA**		**Human**	**Mouse**
ERα	qPCR	Hs00607062_gh	Mm00433143_m1
ERβ	qPCR	Hs01100353_m1	Mm00442411_m1
TGFβ	qPCR	Hs00998133_m1	Mm01178820_m1
Collagen type I	qPCR	Hs00164004_m1	Mm00801666_g1
IGFR	qPCR	Hs00609566_m1	
18s	qPCR	Hs99999901_s1	Mm03928990_g1
**microRNA**		**Forward**	**Reverse**<br>**universal primer for all**
Let-7d		GGGACGAGAGGTAGTAGGTTGC	
29a-5p		TGATTTCTTTTGGTGTTCAGAAA	
34a-5p		GGCAGTGTCTTAGCTGGTTGTAAAA	
142-3p		CGCCATGTAGTGTTTCCTACTTT	
199a-3p		CAGTAGTCTGCACATTGGTTAAAAA	
181b-5p		CGACGACGAACATTCATTGC	
U6		GCAAATTCGTGAAGCGTTCC	
**Antibodies**		**Company**	**Catalog number**
CD63		System Biosciences	Exoab-cd63A-1
pAKT		Cell Signaling	9271
AKT		Santa Cruz Biotechnology, Inc.	Sc-1619
c-jun		Santa Cruz Biotechnology, Inc.	Sc-74543

Caveolin-1	Cell Signaling	3267
ERα	Santa Cruz Biotechnology, Inc.	Human (H184) sc-7207<br>Mouse (MC-20) sc-542
SPC	Abcam	Ab40879
α-SMC	Abcam	Ab8211
Dapi containing mounting medium	Vectorshield	H-1200
