# Supplementary figures and images for: Urine-derived exosomes from individuals with IPF carry pro-fibrotic cargo
Source: eLife. 2022 Dec 1;11:e79543. doi: 10.7554/eLife.79543 (PMC9714968; doi:10.7554/eLife.79543)

Full unedited gels for Fig6 panel G

sample 140 did not show up on pAKT gel

Blot shows 143, 138 and 139

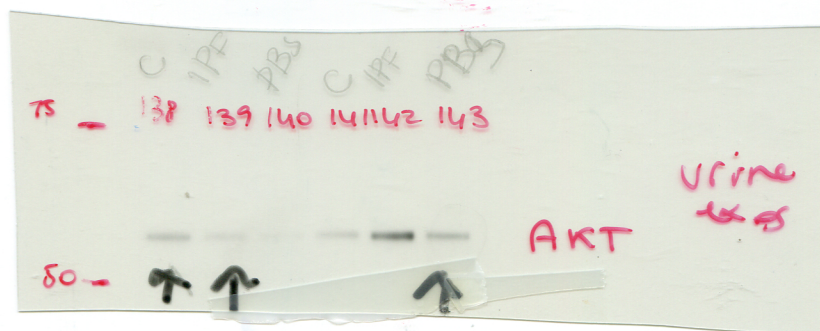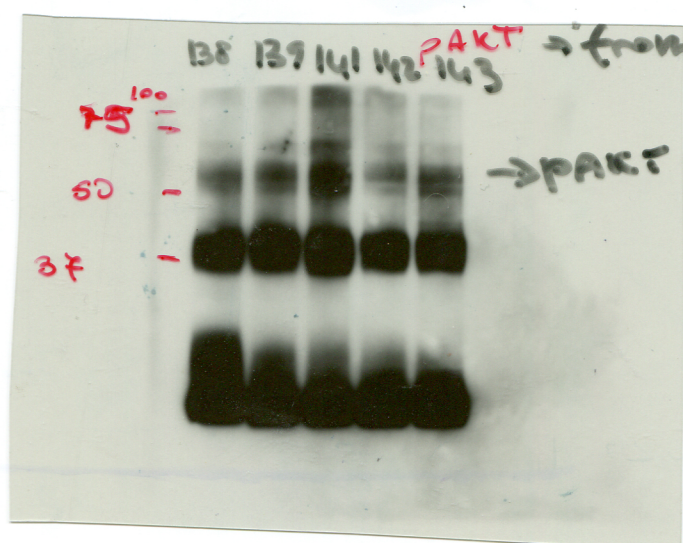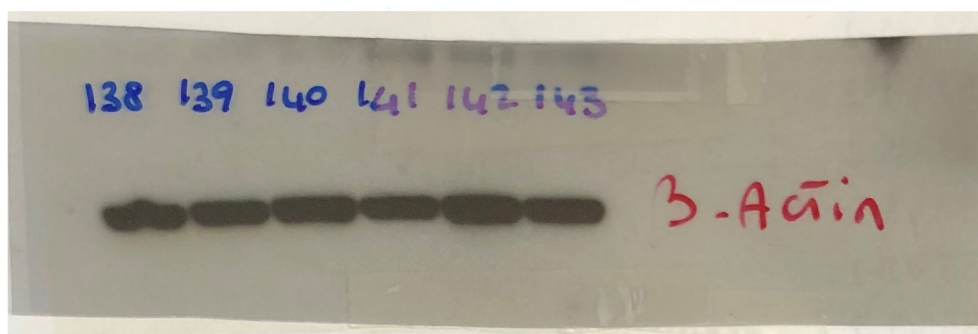

Supplement: Source data 2. [file elife-79543-data2.zip › Raw undedited gels source file/Fig 6G akt full gels.pdf]

C-jun ?

lys Kde  
S. gme.

50 - control IPF PBS

37 -

Figure 6 panel F  
used lanes 1,4 and 5 in figure

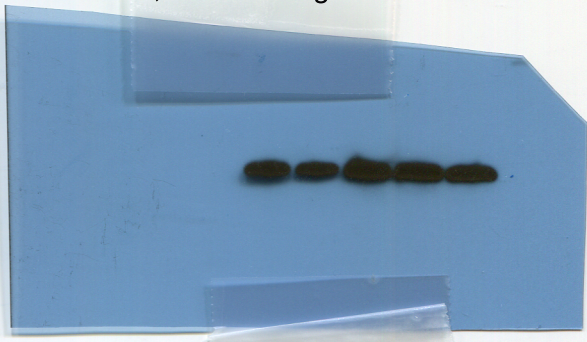

Supplement: Source data 2. [file elife-79543-data2.zip › Raw undedited gels source file/Figure 6F cjun full gel.pdf]

Figure 6 panel H

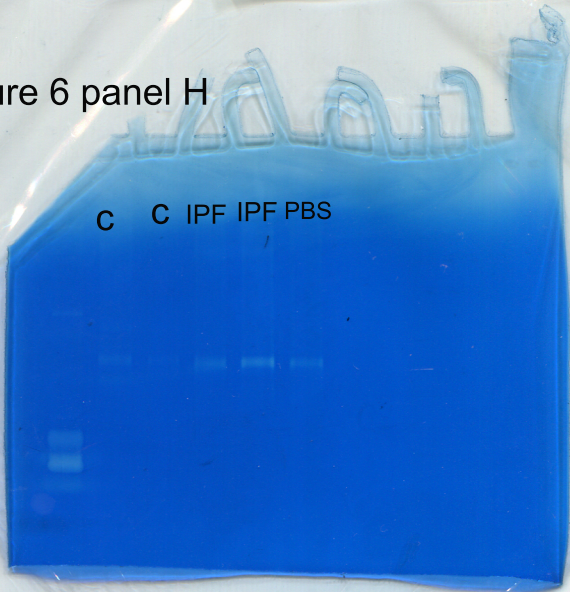

Supplement: Source data 2. [file elife-79543-data2.zip › Raw undedited gels source file/Figure 6H MMP full gel.pdf]

# Full unedited gel for figure 6K

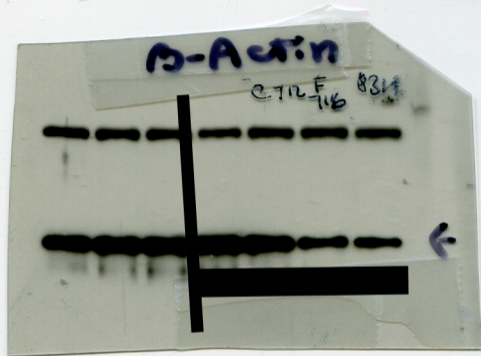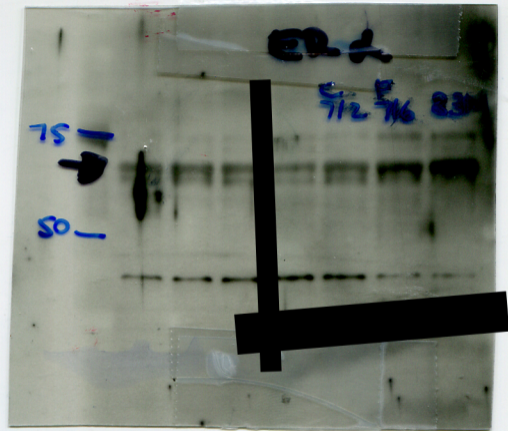

Supplement: Source data 2. [file elife-79543-data2.zip › Raw undedited gels source file/Figure 6k ERa full gel.pdf]

PBS

Control

IPF

Figure 6 panel L

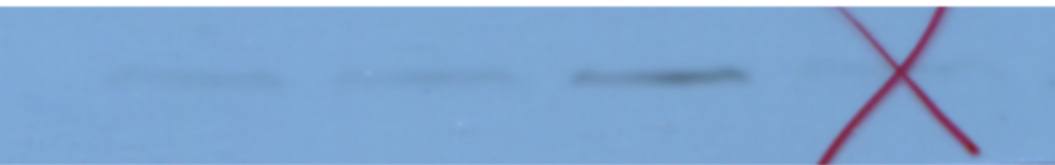

C-Jun

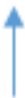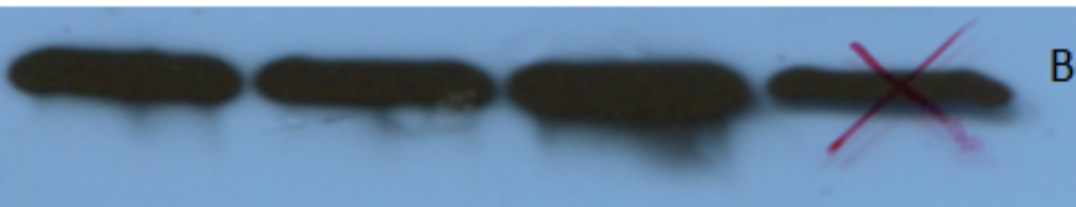

Beta-Actin

Supplement: Source data 2. [file elife-79543-data2.zip › Raw undedited gels source file/Figure 6L cjun full gel.pdf]

Full unedited gel for figure 6L

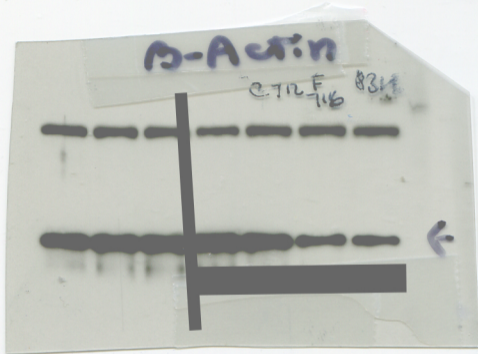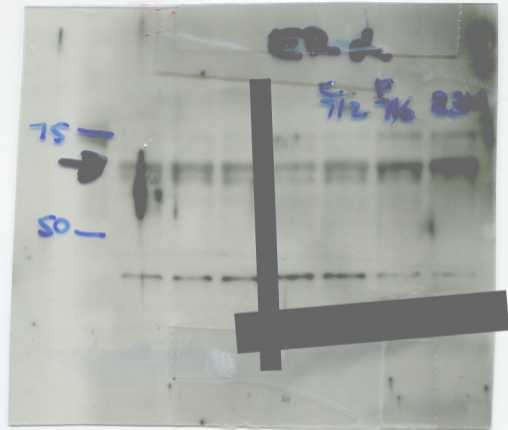

Supplement: Source data 2. [file elife-79543-data2.zip › Raw undedited gels source file/Figure 6L ERa fibroblast full gel.pdf]

PBS

Control

IPF

Panel 6M

C-Jun

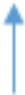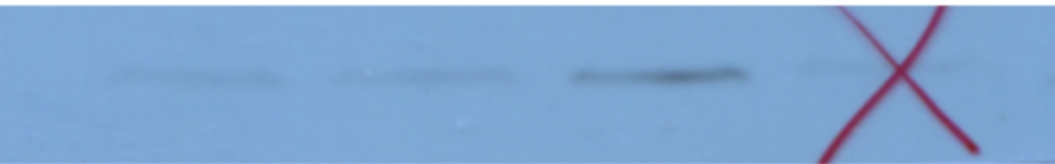

Beta-Actin

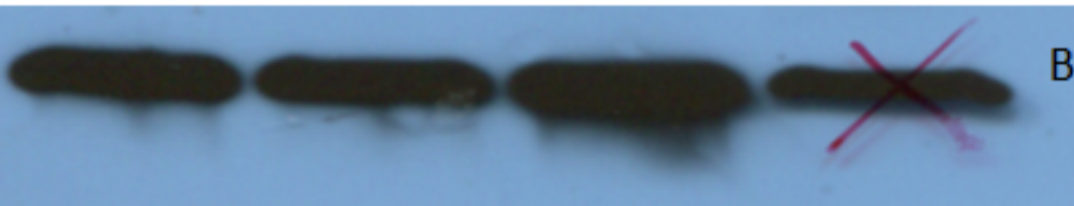

Supplement: Source data 2. [file elife-79543-data2.zip › Raw undedited gels source file/Figure 6M cjun fibroblast full gel.pdf]
